# Deep Eutectic Solvent as Dual Solvent–Catalyst for Efficient Starch Esterification With Vinyl Laurate via Reactive Extrusion

**DOI:** 10.1002/cssc.71087

**Published:** 2026-09-27

**Authors:** Samar Bouzidi, Amira Najahi, Jean‐Marc Lévêque, Patricia Chaud, Frédéric Hugenell, Albert Magnin, Sonia Molina‐Boisseau, Jean‐Luc Putaux, Sami Boufi

**Affiliations:** ^1^ University of Sfax–LMSE–Faculty of Science Sfax Tunisia; ^2^ University Grenoble Alpes, CNRS, Grenoble INP, LRP Grenoble France; ^3^ University Grenoble Alpes, CNRS, CERMAV Grenoble France

**Keywords:** deep eutectic solvent, esterification, reactive extrusion, starch, vinyl laurate

## Abstract

Fatty starch esters have attracted increasing interest as sustainable polymers due to their processing versatility and wide range of applications in biobased and biodegradable materials. However, conventional esterification methods often require highly polar organic solvents, operate under low starch concentrations, and require extended reaction times. We have developed a green approach to esterify wheat starch by reactive extrusion using a novel deep eutectic solvent (DES) based on a mixture of benzyltriethylammonium chloride (BTEAC) and imidazole (IM). This DES acted both as a starch solvent and as an activating agent for vinyl laurate (VL) via the in situ formation of a highly reactive lauryl imidazolide species. The esterification was completed within 15–30 min depending on the reaction temperature. The modification of starch over a broad range of VL/glucose unit molar ratios (0.5 to 2.5) was confirmed using infrared (FTIR) and nuclear magnetic resonance (NMR) spectroscopy analyses that revealed the role of IM in the esterification process, allowing us to detail the reaction pathway. This novel modification approach has not been reported so far and opens new opportunities for the development and upscaling of biobased polymers based on esterified starch, with promising applications in sustainable packaging.

## Graphical abstract

A green approach to esterify wheat starch by reactive extrusion using a novel deep eutectic solvent has been developed. The DES acted both as a starch solvent and as an activating agent to complete the esterification within 15–30 min. This novel approach opens new opportunities for the development and upscaling of biobased polymers with promising applications in sustainable packaging.

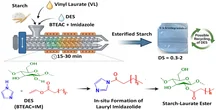

## 1 Introduction

Today, most of mankind's daily activities are carried out using plastics produced from fossil resources because they are easy to synthesize and inexpensive, and most of them have the necessary mechanical properties and thermal stability, enabling countless applications. The worldwide production of plastics derived from fossil resources jumped from 100 million tons in 1990 to about 430 million tons in 2024 [1]. However, the inherent long‐term stability of plastics is a double‐edged sword as they accumulate in the environment if not properly recycled or disposed of Geyer et al. [2] calculated that 80% of ever‐produced plastics from fossil resources have not been properly recycled or disposed of and can be found in landfills, dumps, and the environment. The impact of their presence in the air, soil, and water of our living environment everywhere on Earth has come to light in the last decade, and finding more sustainable alternatives is now a vital necessity for our planet and living species, including mankind. Consequently, using renewable resources to manufacture bioplastics to replace fossil‐based plastics is a desirable first step towards a more sustainable society.

Natural polymers such as proteins or polysaccharides are obvious candidates as renewable resources to replace fossil ones. Among them, starch is probably one of the most attractive since it is available in virtually infinite amounts, as it can be easily isolated from numerous plant sources (potatoes, rice, maize, peas, wheat, etc.) in which it acts as an energy storage material. The botanical source greatly influences the amylose/amylopectin ratio in native starch granules, with both significantly differing in structure, molecular weight, and properties. Although native starch possesses several attractive properties that make it a promising candidate for bioplastics, including its nontoxicity, low cost, abundance, thermal stability, and biodegradability, it also exhibits significant limitations. In particular, its high hydrophilicity, inherent brittleness, and nonfusible nature, decomposing before melting, prevent the direct use of native starch for the development of bioplastics. In particular, its high water uptake negatively impacts its mechanical properties, and the modification of starch into a more hydrophobic material is crucial to avoid any evolution of mechanical properties with relative humidity. One of the oldest methods to counterbalance these drawbacks is chemical modification, in particular, the esterification of the hydroxyl groups by grafting short‐chain carboxylic acids and derivatives [3, 4, 5, 6].

The first reported esterification of starch dates back to 1865. However, the mechanical properties of materials made of starch esterified with short‐chain carboxylic acids (and derivatives) still needed considerable improvement before reaching a potentially interesting industrial scale. Carboxylic acids bearing C4–C8 alkyl chains have progressively been used, and fatty acids bearing C12–C18 moieties have been considered, significantly improving targeted physicochemical properties. To date, vinyl esters are among the most appropriate esterification agents, particularly vinyl laurate and stearate [3, 6, 7]. This was confirmed by our preliminary screening of potential esterifying agents (methyl hexanoate, octanoic acid, succinic anhydride, and vinyl laurate). The esterification was only successfully achieved with succinic anhydride and vinyl laurate (VL), the latter exhibiting superior esterification efficiency under identical experimental conditions (Table S1 and Figure S3). Fatty acid starch esters (FASEs) are emerging as promising biodegradable thermoplastics, readily processable via conventional melt processing, provided their degree of substitution (DS) surpasses a critical threshold determined by the length of the hydrocarbon chains [6, 8, 9]. However, several critical challenges must be addressed before fatty acid starch esters can be produced on a larger scale. Chief among these is the need for more precise control of the DS to better tune the resulting mechanical properties. Additionally, the widely used DMSO solvent for starch esterification should be replaced with a more environmentally friendly alternative that is nontoxic and easily recyclable. The potential for upscaling also requires the development of continuous processing methods with acceptable starch concentrations.

Since the beginning of this century, some new classes of neoteric solvents, namely, ionic liquids and deep eutectic solvents (DESs), have offered greener alternatives to the use of classical organic solvents, thanks to some remarkable and tunable physicochemical properties in many sectors of chemical sciences, including (but not limited to) synthetic chemistry, environmental chemistry, catalysis, physicochemical chemistry, and so on. The chemical modification of starch‐based materials is no exception, and for several years, numerous research groups have reported the beneficial use of these neoteric solvents to chemically modify starch under milder but also greener conditions. Whereas ionic liquids were first tested, DESs have recently shown promising features for the dissolution of carbohydrates, with the advantages over ionic liquids of being inexpensive, easy to prepare, and improving biodegradability, considering that most of them are prepared from natural products. In particular, Zdanowicz et al. [10, 11] have found that imidazole‐based DESs were much more adequate than acidic DESs, facilitating St dissolution and plasticization before further reactions while preserving the structural integrity of starch macromolecules. In addition, it has been hypothesized that the presence of hydrophobic interactions with the dissolving medium was one of the driving forces for improving carbohydrate solubility, which remains an obstacle, in particular when considering the reactivity in the liquid phase, where dissolution time and required temperature may be detrimental to successful esterification [12, 13, 14]. This has since been confirmed by substituting the choline chloride hydrogen bond acceptor (HBA) with benzyltriethylammonium chloride (BTEAC), forming a new subfamily of DESs where, for the same hydrogen bond donor (HBD) compound, the dissolution of carbohydrates was faster, emphasizing the existence of reinforced London forces between the carbohydrates and the DES moieties [15, 16, 17]. Although DESs have been shown to have the potential to replace DMSO as a dissolving solvent [10, 18, 19]. The highly increasing viscosity with increasing amounts of dissolved carbohydrates prevents obtaining the high DS necessary to significantly impact the physicochemical properties of the modified starch. It is possible to overcome the viscosity problem by working at high temperatures with harsh mixing, but the costs and environmental footprint would be too high to consider large‐scale production. Reactive extrusion can be an appropriate answer to overcome these obstacles, as it allows the input of mechanical and thermal energy to facilitate and accelerate dissolution and reactivity in the liquid phase, although the mechanism remains unclear.

The present work proposed to elucidate the mechanism of the esterification of native starch with VL as esterifying agent by reactive extrusion in the presence of a newly designed DES, namely, BTEAC/imidazole (BTEAC/IM). The impact of various processing parameters, such as the method of mixing and initial ratios of starch, DES, and VL, as well as the temperature, on the DS of the resulting starch–laurate esters and characterization of their moisture uptake and hydrophobic character has been studied. A mechanism of St esterification with VL in BTEAC/IM was proposed, and the hypothesis of the dual action of IM as cosolvent and activating agent was demonstrated. To our knowledge, it is the first to report on the preparation of a starch–laurate ester in BTEAC/IM, DES, under reactive extrusion, with an emphasis on the beneficial effect of the DES on the esterification mechanism.

## 2 Experimental Section

### 2.1 Materials

Wheat starch TB (St), containing ca. 20 wt% amylose was kindly provided by Roquette (Lestrem, France). Imidazole, benzyltriethylammonium chloride, vinyl laurate (ethenyl dodecanoate), dimethylsulfoxide, dimethylformamide (DMF), and tetrahydrofuran (THF) were purchased from Sigma–Aldrich (France). All chemicals and solvents were of analytical grade and used without further purification.

### 2.2 Preparation of Deep Eutectic Solvents

The deep eutectic solvents (DES) were prepared by mixing BTEAC and IM at a weight ratio of 1:2.5 in a flask under a gentle flow of argon to prevent moisture uptake, followed by heating at 90°C for 1 h to obtain a homogeneous, colorless, transparent liquid.

### 2.3 Esterification of Starch by Reactive Extrusion

The esterification was performed by reactive extrusion of the DES/St/VL mixture at a constant temperature ranging from 90 to 130°C using a corotating DSM‐Xplore15cc Micro Compounder twin‐screw extruder (TSE), equipped with a clamshell barrel with a conical twin‐screw, 30 cm in length. The screw speed was constant (150 rpm), and, when needed, samples of the extruded material were drawn out through a 2 mm diameter die nozzle for measurement. Three processing routes were tested: (1) After mixing VL, DES, and St for 1 min using an Ultra‐Turrax homogenizer, the extrusion was accomplished at a constant temperature for 1 h. (2) St was mixed with the DES with an Ultra‐Turrax and extruded for 20 min at 90°C until the complete dissolution of St (based on the change in the visual aspect of the extruded mixture from opaque white to a viscous transparent material), followed by the addition of VL and extrusion for 30 min. (3) This route was similar to the second one, with the difference that a 1/1 (molar ratio) VL/IM mixture was added instead of VL alone. All extrusion tests were run at a DES/St ratio of 70/30 (wt%), and the added VL was quantified based on the VL/glucose unit (VL/GU) molar ratio.

Esterified St was recovered by adding ethanol to the extruded product, which was then homogenized by vortex mixing. The precipitate was recovered by centrifugation at 2000 rpm (766 g) for 5 min and washed/centrifuged twice with ethanol to remove any unreacted VL and DES residues. Finally, it was dried overnight at 50°C to remove residual ethanol.

### 2.4 Film Preparation

A sample of esterified St, recovered by precipitation in ethanol, was dissolved in THF or a 70/30 THF/DMF mixture at a concentration of 10 wt% by stirring at 75°C for 1–2 h until complete polymer dissolution, as indicated by the transparency of the solution. The resulting solution was then cast onto a Teflon mold and allowed to dry at 40°C for 1 h, followed by overnight drying at 70°C to ensure total solvent removal.

### 2.5 Characterization

#### 2.5.1 Optical Microscopy

Droplets of suspensions were deposited on a glass slide, covered with a glass slip, and observed between crossed polars with a Zeiss Axiophot 2 microscope equipped with polarized light. Images were recorded with an Olympus SC50 digital camera.

#### 2.5.2 Fourier‐Transform Infrared Spectroscopy (FTIR)

The FTIR spectra were recorded in absorbance mode on a Perkin‐Elmer instrument equipped with a diamond Attenuated Total Reflectance (ATR) accessory.

#### 2.5.3 Thermogravimetric Analysis (TGA)

TGA measurements were performed using a Perkin‐Elmer TGA 800 instrument. The samples (approximately 5 mg) were placed in an alumina crucible and heated from 25 to 800°C under airflow with a heating rate of 10°C min^−1^. The weight loss of the sample was evaluated to determine thermal degradation.

#### 2.5.4 Water‐Contact‐Angle Measurements

Contact angle measurements were performed by depositing calibrated liquid droplets on the surface of the cast films and recording the image of the droplets over time using an OCA 15 Drop Shape Analyzer from Dataphysics. The instrument was equipped with a high‐resolution Charge‐Coupled Device (CCD) camera that acquired 50 images per second, and the data were analyzed with OCA software. All films were conditioned for 1 day at 50% relative humidity (RH) for moisture equilibrium before measurements.

#### 2.5.5 X‐Ray Diffraction (XRD)

Samples were taken after extrusion, dissolved in DMF, then precipitated, and washed with ethanol to remove residual DES and reactants. Thin slices of the resulting solid residues (ca*.* 0.7‐mm thick) were cut, taped on a collimator, and X‐rayed in transmission and under vacuum using a Philips PW3830 generator operating at 30 kV and 20 mA (Ni‐filtered CuKα radiation, *λ* = 0.1542 nm). Two‐dimensional diffraction patterns were recorded on Fujifilm imaging plates and read with a Fujifilm BAS 1800‐II bioimaging analyzer, and the patterns were rotationally averaged to produce diffraction profiles.

#### 2.5.6 Nuclear Magnetic Resonance Spectroscopy (NMR)

^1^H and DEPTQ ^13^C liquid‐state NMR spectra of samples solubilized in DMSO–d6 were recorded with a Bruker Avance 400 spectrometer operating at a frequency of 400.13 MHz for ^1^H and 100.618 MHz for ^13^C. The residual peaks of DMSO–d6 were used as internal standards at 2.5 ppm for DMSO–d6 for ^1^H and 39.51 ppm for DMSO–d6 for ^13^C NMR. The ^1^H spectra were recorded with a 4000 Hz spectral width, 32 000 data points, a 4.09‐s acquisition time, a 1‐s relaxation delay, and 8 scans. DEPTQ spectra were recorded using 90° pulses, a 20,161 Hz spectral width, 65,536 data points, a 1.62‐s acquisition time, a 2‐s relaxation delay, and 3000 scans. For solid‐state NMR, samples were ground into powders, and spectra were recorded at a ^13^C frequency of 100.6 MHz using cross‐polarization (CP) and magic‐angle spinning (MAS), with a 12 kHz spinning speed, a sweep width of 29 761 Hz, and a recycle delay of 2 s. Each spectrum was averaged over 6000 scans. The ^13^C chemical shifts were calibrated with glycine (carboxyl group at 176.03 ppm). Data were processed using Bruker Topspin software.

#### 2.5.7 Degree of Substitution (DS)

The determination of DS can be done by hydrolysis/saponification of the ester bond and back‐titration by NaOH, as it is an easy procedure to follow. However, since the accuracy of this method is disputable, we determined the DS from ^13^C Cross‐Polarization/Magic‐Angle‐Spinning Nuclear Magnetic Resonance (CP/MAS) NMR spectra using the formula:

(1) $$\mathrm{DS}=\frac{A}{C/6}$$

where *A* is the area of the C═O peak of the grafted lauryl at 173 ppm, and *C* is the area of the carbon peaks of the GU unit (C1 to C6) between 58 and 108 ppm.

## 3 Results and Discussions

### 3.1 Aspects of the Starch Esterification With VL in DES by TSE

#### 3.1.1 Dissolution of Starch Granules in DES and DES/VL

The dissolution efficiency of DES was explored by polarized light optical microscopy (Figure 1). Observations were made just after mixing starch granules and DES (*t* = 0) and after 10‐ and 20‐min extrusions at 120 °C. Immediately after mixing, a strong birefringence of individual granules, characterized by the typical Maltese cross, was observed. After 10 min of extrusion, only a limited number of birefringent granules remained visible, and by 20 min, the birefringence was completely lost, indicating the total disruption of the molecular organization in the starch granules by the chemical gelatinization induced by DES. This result confirmed the high solvation efficiency of DES and its capacity to break down the strong intramolecular hydrogen‐bonding network in St granules. This effect was further amplified by the intense shear forces during TSE. The efficient dissolution of St in the DES was also confirmed by the high transparency of the viscous 20 wt% St solution extruded, as shown in Figure S1. The high efficiency of DESs in dissolving starch and disrupting its crystalline and double‐helical structures has also been previously reported in the literature [20, 21]. The addition of VL to DES/St up to a VL/GU = 2/1 (molar ratio) during TSE did not appear to alter the solvation efficiency of DES, as confirmed by the total disappearance of birefringence after a 20‐min extrusion (Figure 1B).

**FIGURE 1:**
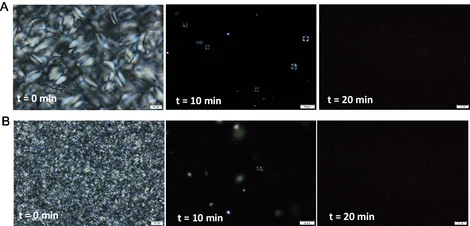
Polarized light optical micrographs of wheat starch granules in the DES (at DES/St ratio of 70/30 wt%) before extrusion (*t* = 0) and after 10‐ and 20‐min extrusions at 120°C, in the absence (A) and in the presence (B) of VL (VL/GU = 2).

#### 3.1.2 Impact of the Mixing Procedure

Preliminary tests of the esterification of St in BTEAC in the presence of VL under reactive extrusion indicated the success of esterification at a temperature between 90 and 120 °C, confirmed by the emergence of an ester band at 1740 cm^−1^ in the FTIR spectra. However, the comparison of the FTIR spectra indicated that the processing route (1), which consisted of simultaneously extruding the VL/DES/St premixed mixture by Ultra‐Turrax, achieved a significantly higher DS. This was evidenced by the higher intensity ratio of C═O absorbance at 1740 cm^−1^ to St absorbance at 1020 cm^−1^ (*A*_C═O_/*A*_St_) (Figure 2B).

**FIGURE 2:**
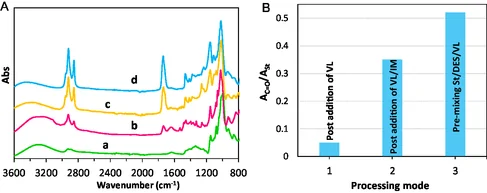
Effect of the processing mode on the efficiency of the esterification of St with VL (VL/GU = 2): (A) FTIR spectra as a function of the mode of addition (a: St, b: postaddition of VL, c: post addition of an IM–VL mixture, d: direct extrusion of the St/DES/VL premix); (B) corresponding absorbance ratios of the A_C═O_ and A_St_ bands.

In addition to achieving the most extensive esterification, the premix route yielded a translucent and viscous extruded material that maintained its appearance over an extrusion time of 1 h (Figure S2). In contrast, the postaddition of VL resulted in a significant fraction of VL remaining immiscible in the DES/St mixture, causing phase separation immediately upon exiting the extruder. The underlying cause of this difference will be addressed later in the discussion of the esterification mechanism. In the following, the esterification reaction was carried out in the presence of VL exclusively using the premixing mode.

#### 3.1.3 Esterification Kinetics and Effect of the VL/GU Ratio

Figure 3A,B presents the FTIR spectra according to processing time and VL/GU ratios, whereas Figure 3C,D displays the relationships between *A*_C═O_/*A*_St_ and time and VL/GU *A*_C═O_/*A*_St,_ respectively. The evolution of the *A*_C=O_/*A*_St_ ratio over time (Figure 3C) for a composition with VL/GU = 2 showed a rapid intensity increase of the C═O band within 30 min, which stabilized over the following hour, indicating the leveling of esterification. This reaction time was notably shorter than that reported for starch esterification in dimethylsulfoxide (DMSO), where 4–5 h were needed to reach a plateau in the DS value despite the use of a catalyst and a lower reagent concentration (10 wt%) [6]. This result indicates that the esterification of St with VL at high solid content (30 wt% based on the St and DES mixture) was effectively achieved using TSE, which is adequate for managing the extremely high viscosity of the mixture while ensuring the thorough homogenization of the reagents. The impact of the VL/GU ratio also has a strong effect on the issue of the esterification reaction, where an increasing intensity of the *A*_C═O_/*A*_St_ ratio can be clearly noticed, as displayed in Figure 3D.

**FIGURE 3:**
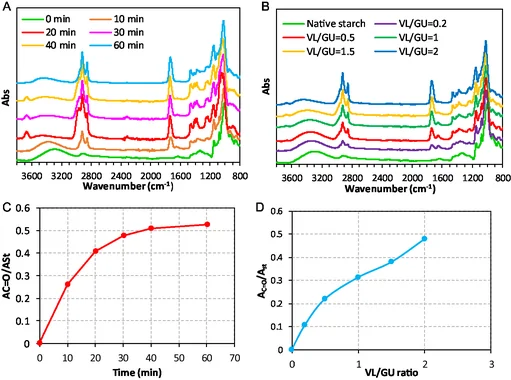
FTIR spectra of the esterified St (A) at different extrusion times (VL/GU = 2) and (B) different VL/GU molar ratios at 120°C using TSE. The corresponding ratio of absorbance of the C═O to St bands for A (C) and B after 30 min (D).

The visual appearance of the extruded product changes with the VL/GU molar ratios, as shown in Figure S2. At 120°C, the St/DES‐extruded product was fully transparent to translucent. In contrast, the St/DES/VL‐extruded mixture transitioned from translucent to opaque, depending on the VL/GU molar ratio. For VL/GU ratios below 1.5, the mixture was translucent, while at ratios above 2, it became white and opaque (Figure S2). This change in optical properties was attributed to variations in the solubility of esterified starch in DES.

Figure 3B shows the FTIR spectra of the esterified St at different VL/GU ratios at 120°C using TSE. The effect of the VL/GU molar ratio on the extent of esterification was examined by following the *A*_C═O_/*A*_St_ ratio according to VL/GU from 0.2 to 2 (Figure 3D). In this study, the St/DES ratio was held constant at 30/70 (wt%), while the amount of VL was progressively increased. As expected, the degree of esterification increased with the VL/GU ratio. However, the trend shifted based on the ratio: a linear increase with a higher slope was observed up to a VL/GU ratio of 0.5, after which the slope decreased for ratios between 0.5 and 2. This behavior might result from changes in the medium polarity as the VL concentration increased.

#### 3.1.4 Effect of Temperature

Temperature has a pronounced effect on esterification kinetics via reactive extrusion (Figure 4). For VL/GU = 2, the *A*_C═O_/*A*_St_ absorbance ratio rapidly reached a plateau of approximately 0.5 after 40 min at 120°C, whereas at 90°C, it only reached 0.46 after 150 min. Similarly, for VL/GU = 1.5, *A*_C═O_/*A*_St_ stabilized after 40 min of extrusion at 120°C. Optical observations of the extruded material showed persistent birefringence even after 60 min at 90°C (Figure S3), indicating incomplete dissolution of St under these milder conditions. In contrast, the enhanced dissolution of St in DES at 120°C likely contributes to the faster esterification kinetics. At 130°C, the effect becomes even more pronounced: a steep increase in the *A*_C═O_/*A*_St_ ratio is observed, reaching 0.8 within only 30 min for VL/GU = 2.5. This marked acceleration of the esterification reaction with increasing temperature is highly beneficial in performing the modification of St with VL in DES via reactive extrusion.

**FIGURE 4:**
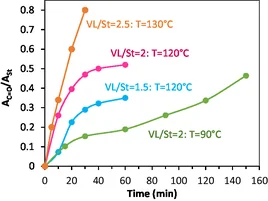
Evolution of the ratio of absorbance of the C═O to St bands with different VL/GU ratios and at different extrusion temperatures.

### 3.2 Characterization of the Starch–Laurate Esters

Esterified St samples with different VL/GU ratios were analyzed by both FTIR (Figure 5A) and solid‐state CP/MAS NMR (Figure 5B) to explore a potential direct relationship between the two analytical methods. The FTIR spectra of native St exhibited typical bands at 3360 cm^−1^ (*ν*_OH_), 2928 cm^−1^ (*ν*_C–H_), 1645 cm^−1^ (*∂*_OH_), 1157 cm^−1^, 2900–3000 cm^−1^ (C─H stretching), 1155 cm^−1^, 1080 cm^−1^, 1020 cm^−1^, 925 cm^−1^, and 900–1100 cm^−1^ (C─OH bending). After esterification, a clear evolution of the spectra was observed, with the emergence of the carbonyl group of the substituted laurate at 1740 cm^−1^ (*ν*_C═O_) and two bands at 2920 and 2860 cm^−1^ attributed to the *ν*_CH_ of the alkyl groups. The intensity of these bands increased with increasing VL/GU ratio. A decrease in the area of the OH band at 3360 cm^−1^ was also seen to a nearly complete disappearance for samples with a VL/GU ratio exceeding 2. We also noted the emergence of a new band around 3700 cm^−1^, clearly visible in the FTIR spectra in Figure 3A, which is attributed to the stretching vibration of free hydroxyl groups of St. This band likely appeared due to the shielding of H─bond interactions among the residual hydroxyl groups of St.

**FIGURE 5:**
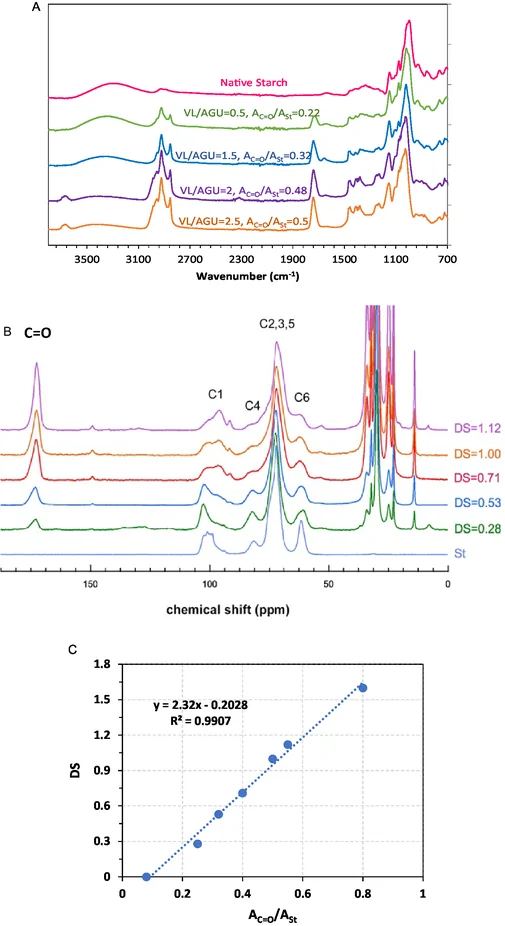
(A) FTIR spectra of native starch (St) and starch esterified with VL in DES at different VL/GU ratios; (B) ^13^C CP/MAS NMR spectra of native St and VL‐esterified St in conditions summarized in Table S2; (C) linear correlation between DS and A_C═O_/A_St_ ratios.

The evolution of the chemical structure of the starch–laurate esters was further studied by liquid‐ and solid‐state ^13^C‐NMR on powdered samples. The ^13^C‐NMR spectra of native and modified St are given in Figure 5B. For native starch, the signals between 60 and 65 ppm were assigned to C6 of the glucosyl unit, while the overlapping signals between 68 and 78 ppm were associated with C2, C3, and C5. The resonance at 82 ppm was assigned to C4 and those at 90 and 105 ppm to C1. In the VL‐esterified starch spectra, the introduction of the lauryl chains resulted in new peaks at 10–35 ppm corresponding to the carbon resonances of the lauryl chains and the C atom of the ester group (O═C─O) (170–175 ppm). With increasing VL/GU ratios, the signal from lauryl chains increases at the expense of the signals from starch, indicating an increase in the esterification of St. In addition to the expected presence of the new peaks enumerated just above, one can note the emerging presence of small peaks at 149 ppm and 52 ppm, respectively. These peaks are likely due to the presumed side reactions; further investigation is currently being conducted to elucidate these unexpected peaks.

After normalization of the CP/MAS spectra of esterified St with varying VL/GU ratios, the DS was measured, and the absorbance intensity ratio of the carbonyl (C═O) band was correlated to that of the St band at 1020 cm^−1^ in the FTIR spectra (*A*_C═O_/*A*_St_). The data presented in Figure 5C indicate a linear correlation between DS and *A*_C═O_/*A*_St_ within the DS range of 0.08–1.60. This correlation allows for a reliable estimation of the evolution of DS during the esterification process. One can notice that the line does not pass through the origin and crosses the *x*‐axis at 0.08. Since native starch was not esterified, the weak C═O absorbance at 1720 cm^−1^ is likely due to the presence of endogenous lipids. Although an acceptable DS of 1.6 can be achieved at a VL/GU ratio of 2.5 and at 130°C, it was not possible to reach a higher DS, even in the presence of a VL/GU ratio of 3 (not shown). Several hypotheses can be made:

1. The precipitation of St occurred when the VL concentration exceeded a critical level. This hypothesis was supported by the observation that at a VL/GU ratio of 2.5, the mixture turned white (Figure S2), whereas it remained translucent at VL/GU < 2.
2. The lauryl chains grafted by esterification may have sterically hindered access to the remaining hydroxyl groups, reducing the efficiency of the esterifying agent.
3. The clustered architecture of amylopectin (the major constituent of the native granules) [22] and specifically the presence of numerous branching points may sterically limit the access of the esterification agent to the short branches of the macromolecule.

However, further investigation is needed to clarify the factors limiting the DS of the St–laurates prepared under our esterification conditions, and experiments at higher reaction temperatures and/or reaction times are in progress.

The resonances associated with the C1 of glucose units underwent a significant evolution upon esterification. The C1 contribution of native starch contains four resonances. Those at 98.9, 100.0, and 101.0 ppm correspond to the A‐type allomorph typical of cereal starch sources, while the peak at 102.4 ppm is related to the amorphous fraction [22]. After extrusion, the A‐type peaks disappeared, reflecting the disruption of the semicrystalline granules. For low esterification degrees, the main peak at 102.6 ppm can be analyzed as the superimposition of an amorphous fraction and the presence of a V‐type inclusion complex between nonesterified amylose and endogenous lipids [23]. The contribution of this peak decreased with increasing DS values, while a new peak at 96.1 ppm increased concomitantly, reflecting a conformational change of C1 of the esterified glucose unit. This resonance has previously been described for highly substituted starch esters [9].

The XRD profile of native wheat starch granules contained five main peaks located at 2*θ* = 15.0°, 17.0°, 17.9°, 19.8°, and 22.9° and corresponded to the A‐type allomorph (Figure 6A) [24]. These peaks were absent in the profiles of the St–laurate esters, indicating that the initial semicrystalline St granules were completely disrupted during the treatments. Three weak peaks located at 7.8°, 13°, 7°, and 20.8° in the profile of the sample esterified at 120°C for 30 min with VL/GU = 0.4 suggest the presence of a V‐type allomorph formed by nonesterified amylose with endogenous lipids, consistent with the corresponding solid‐state NMR spectrum (Figure 5B). The allomorph would be V6_a_, the dry form of V6_I_ that contains 6‐fold amylose single helices incorporating the alkyl moiety of the lipid.

**FIGURE 6:**
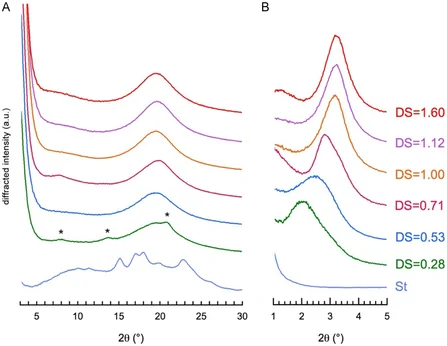
(A) XRD profiles of native St and St–laurate esters prepared under different conditions summarized in Table S2, as a function of DS. The stars indicate the peaks corresponding to the presence of a V‐type complex. (B) Close‐up on the lower‐angle scattering region. The *y*‐scale is different in (A) and (B).

For the tested conditions shown in Figure 6, the broad peaks with a maximum at *ca*. 19.6° illustrated the amorphous character of St laurates, in agreement with the results of Ojogbo et al. [25]. However, a narrower and more intense peak at a lower angle, absent in the profile of St, was observed for all esters. The close‐up of this region in Figure 6B showed that the position of the peak maximum varied depending on the esterification conditions. This low‐angle peak is likely related to the average distance between St laurate chains, which depends on the molecular volume and the number of grafted lauryl chains extending out of the amylose backbone. A similar feature was reported by Crépy et al. [26] for cellulose esters substituted with C8–C18 chains, but the authors proposed that the materials contained a layered structure, which was plausible considering the structural model describing the fully substituted cellulose–laurate chains. In that case, the intermolecular distance increased with increasing substituent length.

In native wheat St granules, amylose is considered to be amorphous, while the short branches of amylopectin are associated into 6‐fold double helices that form A‐type crystallites [24]. However, both amylose and amylopectin become available for chemical modification after disruption and solubilization of the granules. In the case of amylose laurates, amylose also likely adopts a single helical conformation with lauryl substituents extending out of the amylose backbone, forming a brush‐like structure (Figure S6). As demonstrated for a few amylose esters for which molecular models were calculated from crystallographic data, the length and size of the side group influence the helical conformation of the amylose chain. For instance, in amylose triacetate I, the amylose backbone adopts a 14‐fold conformation with a noninteger 4.67 number of residues per helical turn [27]. While in amylose tripropionate, tributyrate, and trivalerate, amylose adopts a 5‐fold conformation [28]. To our knowledge, such a crystallographic model does not exist for amylose trilaurate. In our study, St–laurate esters were amorphous. The DS below 1.2 and likely stereo‐irregularity prevented proper crystallization. (Figure 6). Nevertheless, the geometrical molecular model shown in Figure S6, based on an amylose backbone with 11 glucosyl residues, allowed us to assess the impact of the brush‐like morphology on the intermolecular distance. With a DS of 3, the molecule diameter would be about 3.5 nm, which is also the intermolecular distance between parallel chains when no interdigitation of the lauryl side chains is considered. A partial interdigitation would thus result in a smaller distance, which would further decrease with decreasing DS. The intermolecular distances deduced from the low‐angle peak maximum were 4.36, 3.59, 3.14, 2.80, 2.78, and 2.75 nm for St laurates with DS = 0.28, 0.53, 0.71, 1.00, 1.12, and 1.60, respectively. For 1 ≤ DS ≤ 1.6, the aforementioned assumption was verified, while it was contradicted for DS values lower than 1. Considering the evolution of the C1 resonances of the GUs (Figure 5B), we propose that above a DS of 1, due to the steric influence of the laurate substituents, the C1 of GUs undergoes a conformational change that results in a straightening of the amylose backbone, thus promoting the alignment of St laurate with a shorter intermolecular distance. However, further modeling explorating the influence of DS is necessary to validate this hypothesis.

### 3.3 Mechanism of Starch Esterification in the DES

The sensitivity of the esterification reaction to the mode of VL addition (i.e., one‐pot mixing vs. postaddition) suggests a possible interaction between VL and the DES or one of its constituents, presumably rather with IM than with BTEAC, unexpectedly leading to an increase in reactivity in the esterification process. To verify this hypothesis, VL was mixed with IM separately in different ratios, and the FTIR spectra of each composition were recorded (Figure 7A,B). In the presence of IM, significant changes in the original FTIR spectrum of VL were observed. The intensity of the band at 1650 cm^−1^, corresponding to VL, decreased at IM/VL = 1/1, with the complete disappearance of this band at the 2/1 ratio; (ii) the C═O band of VL shifted from 1760 to 1740 cm^−1^ for IM/VL = 2/1; (iii) the bands at 1660, 1540, and 1480 cm^−1^, which were present in the spectrum of pure IM, disappeared for IM/VL = 2/1; and (iv) new bands emerged at 1050 and 1240 cm^−1^.

**FIGURE 7:**
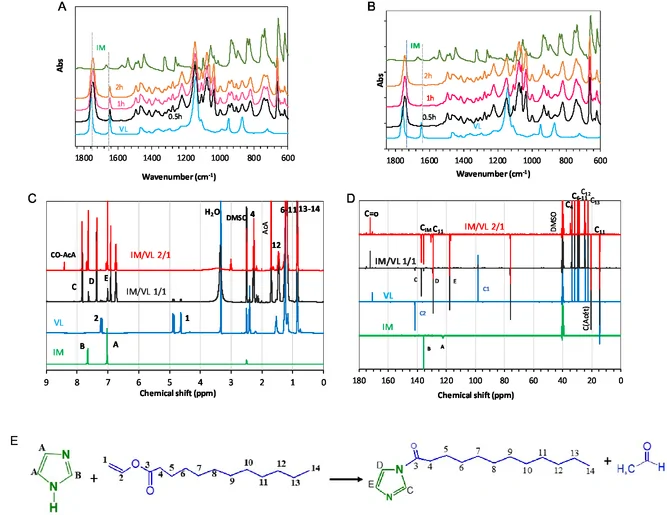
FTIR spectra of IM and IM‐VL mixtures as a function of time: (A) molar ratio IM/VL = 1/1, (B) IM/VL = 2/1. Liquid‐state NMR spectra of IM, VL, and IM/VL mixtures at two molar ratios (1/1 and 2/1): ^1^H (C) and ^13^C (D) spectra recorded in DMSO–d6; E) proposed reaction pathway between IM and VL with the numbering of carbon atoms, drawn with MarvinSketch [29]. AcA and C═O‐AcA refer to the acetaldehyde and C═O group of the acetaldehyde, respectively.

Considering that the band at 1650 cm^−1^ corresponds to the C═C stretching vibration in VL and the 1660 cm^−1^ band to the N─H stretching vibration in IM, we infer that both the vinyl group in VL and the N─H group in IM are lost due to an unexpected side reaction occurring when the two are mixed. This indicates the formation of an N‐acylimidazole species through the condensation of IM with VL, accompanied by the release of acetaldehyde. The reaction occurred within 30 min of mixing. The FTIR spectra showed the complete disappearance of the C═C band at 1650 cm^−1^, with no further spectral changes over time (Figure 7B).

Further confirmation of the reaction between VL and IM was provided by NMR analysis of the IM–VL mixture. To better illustrate the shifts in peak position, the ^1^H and ^13^C NMR spectra of IM, VL, and IM–VL mixtures at 1/1 and 1/2 ratios were overlaid (Figure 7C,D). In the ^1^H NMR spectrum for the 1/1 IM/VL ratio, the peaks corresponding to IM at 7.0 and 7.7 ppm showed a marked intensity decrease along with the appearance of three peaks at 6.9, 7.3, and 7.7 ppm. The signals from VL also shifted, particularly the vinyl protons (CH_2_=CH─) at 4.6, 4.8, and 7.25 ppm, whose intensity significantly decreased at the 1/1 ratio and completely disappeared at the 2/1 ratio. In addition, the resonances of the methylene groups in VL slightly shifted: the methylene group adjacent to the carbonyl group, initially at 2.4 ppm, shifted to 2.25 ppm, while the next methylene group moved from 1.55 to 1.45 ppm. These shifts were most pronounced at a 2/1 IM/VL ratio, where the methylene peaks completely disappeared.

A similar trend was observed in the ^13^C NMR spectra (Figure 7B). The signals from IM at 122 and 135 ppm disappeared and were replaced by three new peaks at 118, 129, and 137 ppm. For VL, the vinyl carbon signals at 99 and 135 ppm disappeared after mixing with IM, and two new peaks appeared at 76 and 20 ppm, corresponding to CH and CH_3_ carbons (based on the analysis of DEPT ^13^C spectra). These changes in the NMR spectra support the conversion of VL to N‐lauryl imidazole upon mixing with IM, which is consistent with the reaction pathway shown in Figure 7D. Additionally, the emergence of new peaks at 76 and 20 ppm in the ^13^C CP/MAS NMR spectra and at 6.75 ppm in the ^1^H NMR spectrum of the VL/IM mixture suggests that a secondary reaction involving the released acetaldehyde may have occurred. One possibility is a reaction between IM and acetaldehyde as shown in Figure 8B. However, from the NMR data, we could not unambiguously confirm the likely reaction occurring between IM and released acetaldehyde.

**FIGURE 8:**
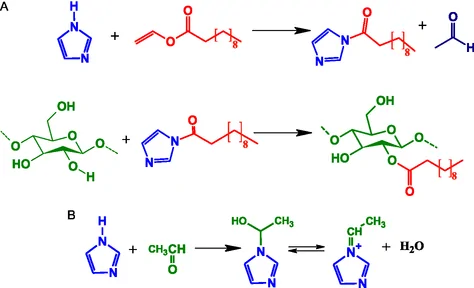
(A) Reaction scheme for starch esterification in DES using VL involving the formation of laurate imidazolide in situ during reactive extrusion; (B) possible reaction between imidazole and generated acetaldehyde.

The interaction between VL and IM sheds light on several discrepancies observed during the reactive extrusion of St in DES in the presence of VL. The fast and successful esterification that was observed when all components (IM, BTEAC, St, and VL) were extruded together can be attributed to the fast conversion of VL into N‐lauryl imidazole (LIM). The latter was not only more reactive than VL but also exhibited better miscibility in the DES solvent. Once formed, LIM quickly reacted with the starch granules, which had been swollen by the DES solvent. As the esterification of St macromolecules progressed, they detached from the granules, exposing the inner starch chains to further reactions with LIM. In contrast, when starch was predissolved in DES, the strong solvation of the St chains by IM and BTEAC reduced the availability of IM for its reaction with VL. This effect was further accentuated by the lipophilic nature of VL, which limited its solubility in the polar St–DES mixture. Based on the aforementioned findings, the proposed mechanism of starch esterification with VL in the DES is illustrated in Figure 8.

The increased reactivity of vinyl laurate‐imidazole compared to singly VL in esterification with alcohols can be explained by two key factors: first, the carbonyl group becomes more electrophilic after the vinyl group is displaced by imidazole, and second, imidazole is an excellent leaving group. *N*‐acylimidazoles are widely used as activating agents in organic synthesis, particularly for the mild and efficient esterification of primary, secondary, and tertiary alcohols. Typically, in organic chemistry, *N*‐acylimidazoles are synthesized via the reaction of carboxylic acids with *N,N*′‐carbonyldiimidazole (CDI) [30, 31]. In this case, however, *N*‐acylimidazole is generated by reacting VL with IM, which results in the release of acetaldehyde. It is worth mentioning that the formation of carboxylic acid imidazolides was reported and demonstrated by Blohm and Heinze [32] during the esterification of starch with propionic anhydride and lauric acid in molten imidazole. In this process, the reaction between propionic anhydride and imidazole rapidly occurred to form the corresponding imidazolide.

### 3.4 Hydrophilicity and Moisture Sorption of the St–Laurate Ester Films

Thin films of St–laurate esters, synthesized at varying VL/GU molar ratios, were prepared via solvent casting. The St–laurate esters were soluble in THF or a THF/DMF mixture when prepared at VL/GU ratios below 1.5, and the resulting films were transparent (Figure 9A). This high transparency was likely due to the amorphous character of the St–laurate esters.

**FIGURE 9:**
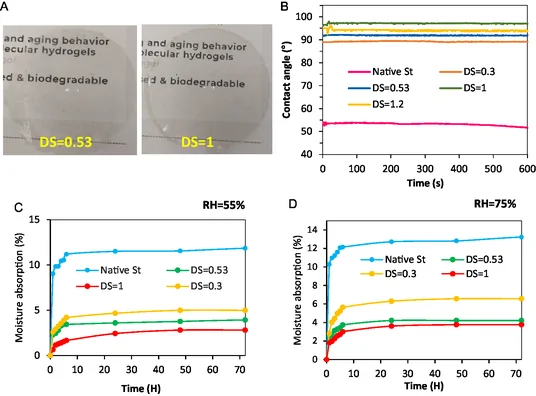
(A) Photographs of the transparent St–laurate films; water contact angle (B) and moisture uptake of neat St and ST–laurate films with different DS, measured at (C) 55% RH and (D) 75% RH.

One of the primary limitations of using St in packaging applications, whether plasticized or blended with other biodegradable polymers, is its high sensitivity to moisture, which adversely affects the mechanical properties of the resulting films as water acts as a plasticizer for starch. To assess the changes in the hydrophilic/hydrophobic character of lauryl‐modified St, water contact angles were measured on thin films prepared by solvent casting of St laurates prepared with various VL/GU ratios. The native St film exhibited a contact angle of approximately 53°, consistent with literature values (45°–65°) depending on the St type and lipid content [33]. Upon esterification with lauryl groups, the water contact angle markedly increased, reaching values between 89° and 98°, depending on the DS (Figure 9B). These values remained stable over time, indicating a hydrophobic surface with no evidence of water sorption. Notably, even at a DS of 0.3, the contact angle reached approximately 90°, demonstrating a significant transition in surface properties from hydrophilic (*θ* ≈ 54°) to hydrophobic. This marked evolution in the surface properties was attributed to two main factors: (i) the substitution of hydroxyl groups in the GUs with hydrophobic lauryl chains and (ii) the formation of an umbrella‐like structure by the long grafted hydrocarbon chains, which effectively masked unreacted hydroxyl groups. This structural arrangement accounts for the relatively high contact angles (around 90°) observed even at low DS.

For samples prepared with VL/GU ratios between 1 and 2, the maximum moisture sorption (*W*_max_) was approximately 2.8 and 4 wt% at 55% and 75% RH, respectively, which is markedly lower than that of native starch, which exhibited *W*_max_ values of around 12 and 17 wt% under the same conditions (Figure 9C,D).

The next step of this work is to investigate the effect of starch esterification with VL in the DES on the processability of the resulting material as well as its suitability for fabrication into films and molded objects using conventional melt‐processing methods. Another important aspect will be to assess how the DS influences the biodegradability of the modified starch.

## 4 Conclusion

We have described an environmentally friendly esterification process of wheat St using VL in a novel basic DES based on BTEAC and IM. This process, conducted via reactive extrusion, operated at high starch concentrations of up to 30 wt% and VL/GU ratios ranging from 0.5 to 2.5. Our results demonstrate that BTEAC/IM effectively solubilizes starch at 90–120°C within 10–20 min, enabling rapid and high‐degree esterification with VL in under 30 min, making the process both efficient and economically viable. Beyond its role as a solubilizing agent, DES also acts as an activating catalyst for VL through its reaction with IM, forming a highly reactive lauryl imidazolide intermediate. The formation of this species was confirmed via FTIR and NMR analysis. The successful esterification of starch was further validated using FTIR, ^1^H NMR, and solid‐state ^13^C CP/MAS NMR, and a DS ranging from 0.2 to 1.7 was achieved when the VL/GU ratio varied between 0.5 and 2.5. The XRD analysis revealed that the starch laurates were amorphous, with the laurate chains adopting a brush‐like architecture, where lauryl substituents extend outward from the amylose backbone in a single‐helical conformation. In addition, esterification with VL significantly reduced the moisture uptake. To our knowledge, this proposed process, described for the first time, is environmentally sustainable, utilizes the nonhazardous BTEAC/IM DES, and is also economically advantageous due to its continuous operation at high starch concentrations up to 30 wt%. Work is in progress to investigate the impact of the DS achieved with VL on the biodegradability of the St–laurate esters and to evaluate their suitability for producing thin films via extrusion‐blowing processing. The recyclability of the BTEAC/IM DES will also be investigated.

## Author Contributions

**Samar Bouzidi:** investigation, formal analysis, writing – original draft. **Amira Najahi:** investigation, formal analysis, writing – original draft. **Jean‐Marc Lévèque:** funding acquisition, project administration, supervision, conceptualization, supervision, formal analysis, writing – review and editing. **Patricia Chaud:** investigation, formal analysis. **Frédéric Hugenell**: investigation, formal analysis. **Albert Magnin:** funding acquisition, project administration, writing – review and editing. **Sonia Molina‐Boisseau:** funding acquisition, writing – review and editing. **Jean‐Luc Putaux:** funding acquisition, writing – original draft, investigation, formal analysis, writing – review and editing. **Sami Boufi:** funding acquisition, project administration, conceptualization, supervision, formal analysis, writing – review and editing.

## Conflicts of Interest

The authors declare no conflicts of interest.

## Supporting information

Supplementary Material

## Data Availability

The data will be made available on reasonable request.
