# Supplementary material for: Deep Eutectic Solvent as Dual Solvent–Catalyst for Efficient Starch Esterification With Vinyl Laurate via Reactive Extrusion
Source: ChemSusChem. 2026 Sep 27;19(19):e71087. doi: 10.1002/cssc.71087 (PMC13617360; doi:10.1002/cssc.71087)
Supplement: Supplementary file 1 [file CSSC-19-e71087-s001.pdf]

Deep Eutectic Solvent as Dual Solvent-Catalyst for Efficient Starch Esterification with Vinyl Laurate via Reactive Extrusion

Samar Bouzidi. Amira Najahi. Jean-Marc Lévêque. Patricia Chaud. Frédéric Hugenell. Albert Magnin. Sonia Molina-Boisseau. Jean-Luc Putaux and Sami Boufi

Supplementary material

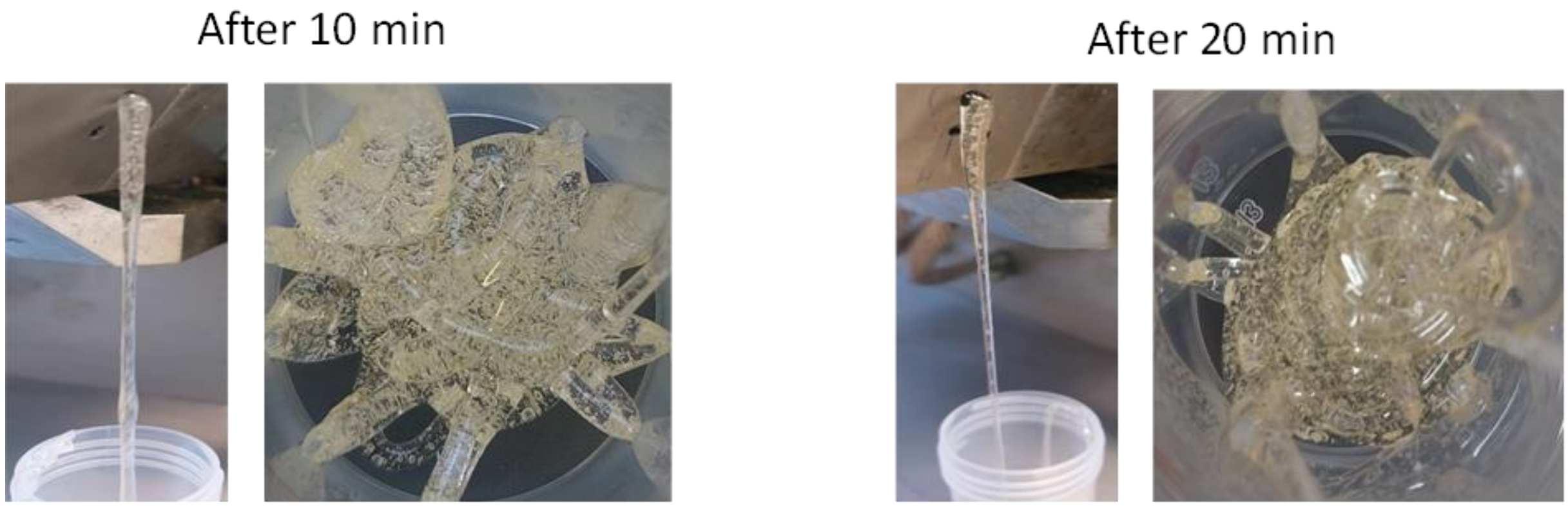

Figure S1. Visual aspect of the extruded starch/DES mixture after 10 and 20 min at 120 °C (DES: BTEAC/IM 30/70 wt%).

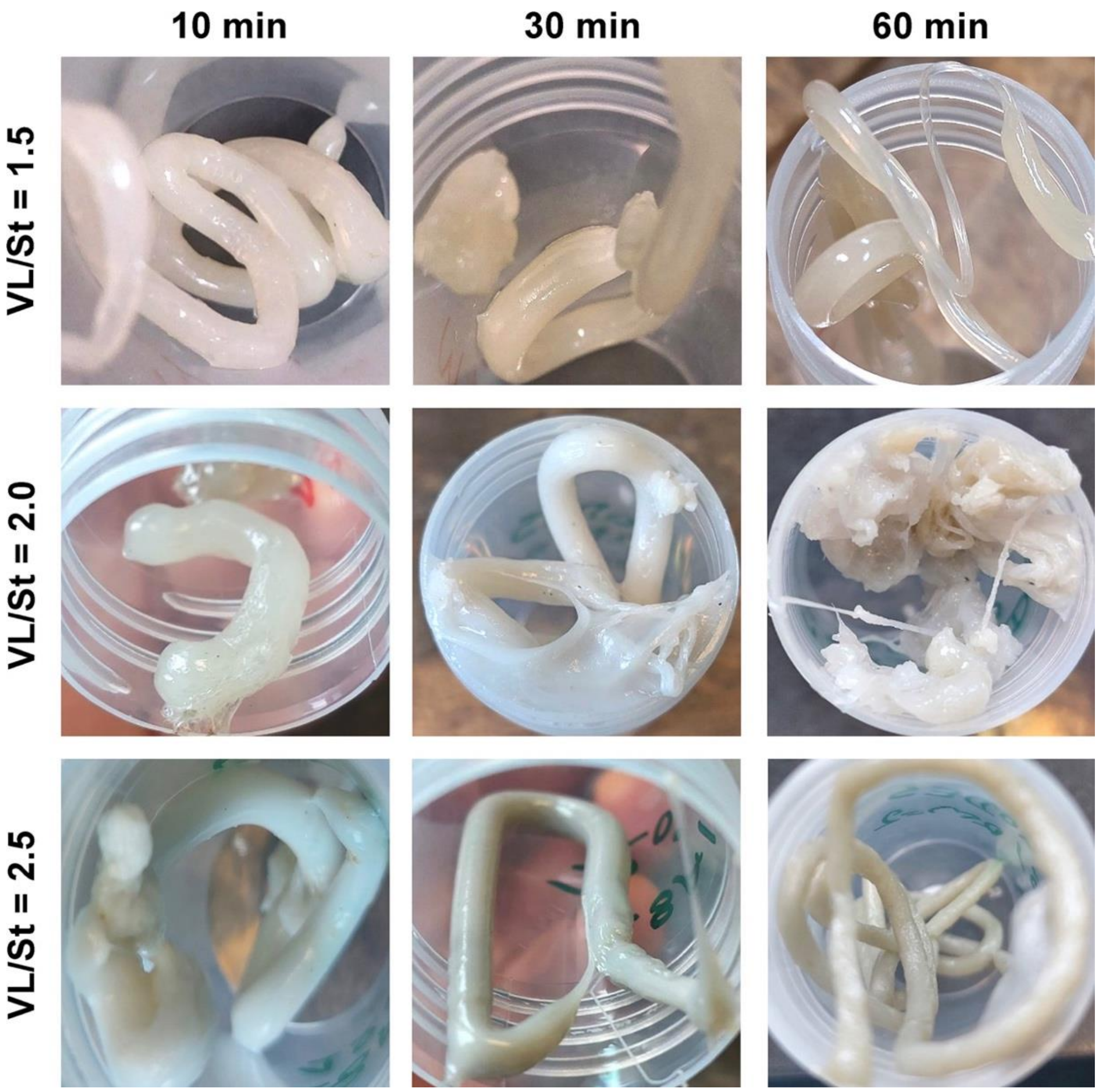

Figure S2. Visual aspect of the extruded product during the esterification of starch with VL at VL/GU=1.5. 2.0. and 2.5. in BTEAC/IM DES (St/DES 30/70 wt%) at 120 °C as a function of extrusion time.

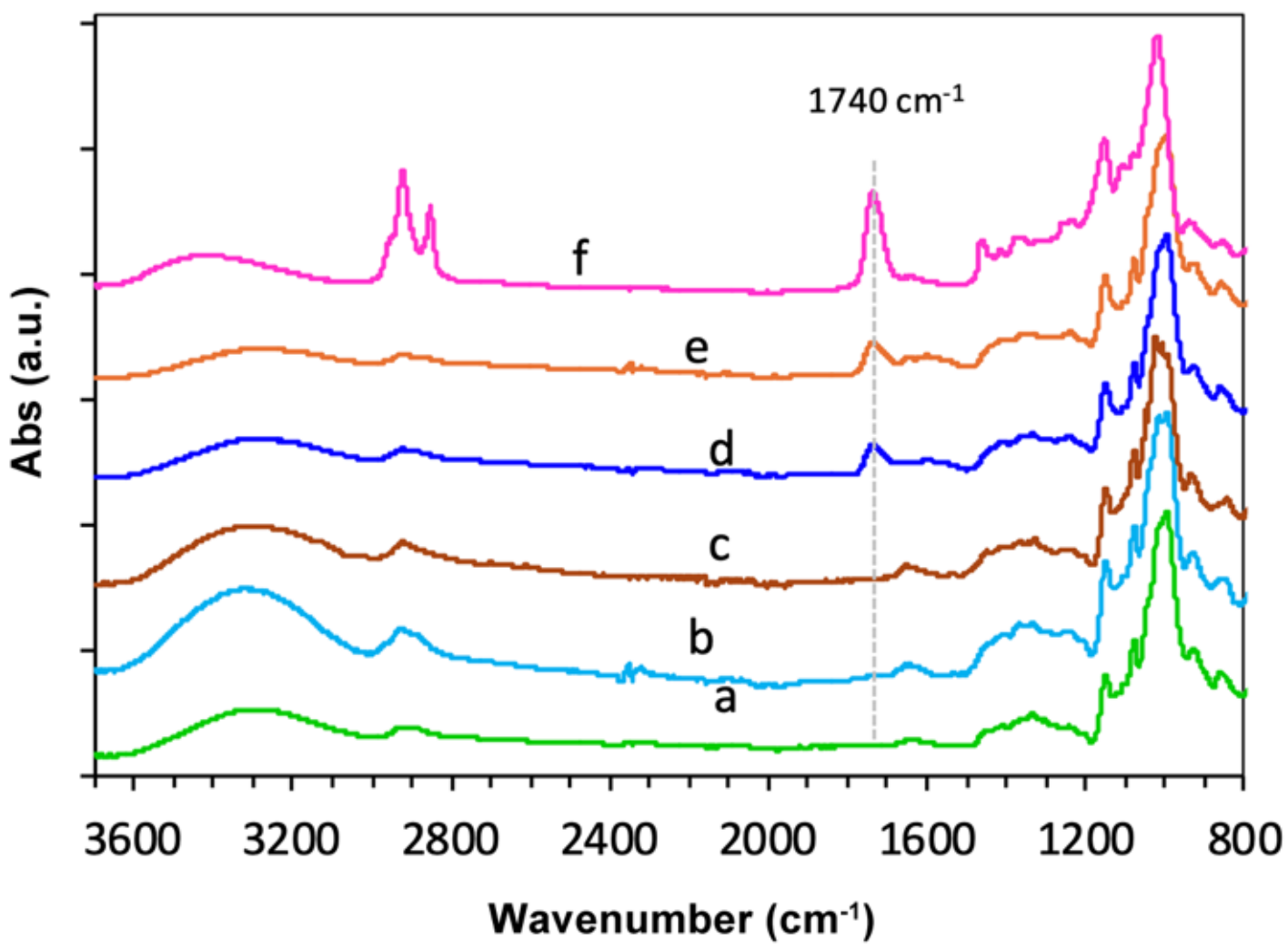

Figure S3. FTIR spectra of neat starch (a). and recovered product after reactive extrusion in DES at 90 °C during 60 min. in the presence of methyl hexanoate (b). octanoic acid (c). succinic anhydride (SA/GU = 1.5) (d). succinic anhydride (SA/GU= 3) (e). and vinyl laurate (VL/GU= 1.6) (f).

Table S1. Preliminary tests for the esterification of starch in DES in the presence of different esterifying agents.

Esterifying agent (EA)	St/DES/EA composition (wt%)	EA/GU molar ratio	Temperature (°C)	Extrusion time (min)	FTIR CO band	FTIR A_{CO}/A_{St}
Octanoic acid	30/50/20	1.00	90	30	No	-
Octanoic acid	30/50/20	1.00	100	60	No	-
Methylhexanoate	20/50/30	1.50	90	60	No	-
Succinic anhydride	30/40/30	1.50	90	60	Yes	0.12
Vinyl laurate	15/45/40	1.50	90	60	Yes	0.30
Vinyl laurate	20/30/50	2.15	90	60	Yes	0.45

Table S2. Effect of VL/GU ratio and processing temperature on ACO/ASt intensity and degree of substitution (DS)

VL/GU	T (°C)	t (min)	A_{CO}/A_{St} IR	DS RMN
-	-	-	*0.08*	*0*
0.40	120	30	0.25	0.28
1.50	120	30	0.32	0.53
2.00	90	60	0.40	0.71
1.67	90	90	0.50	1.00
2.50	120	30	0.55	1.12
2.50	130	30	0.80	1.60

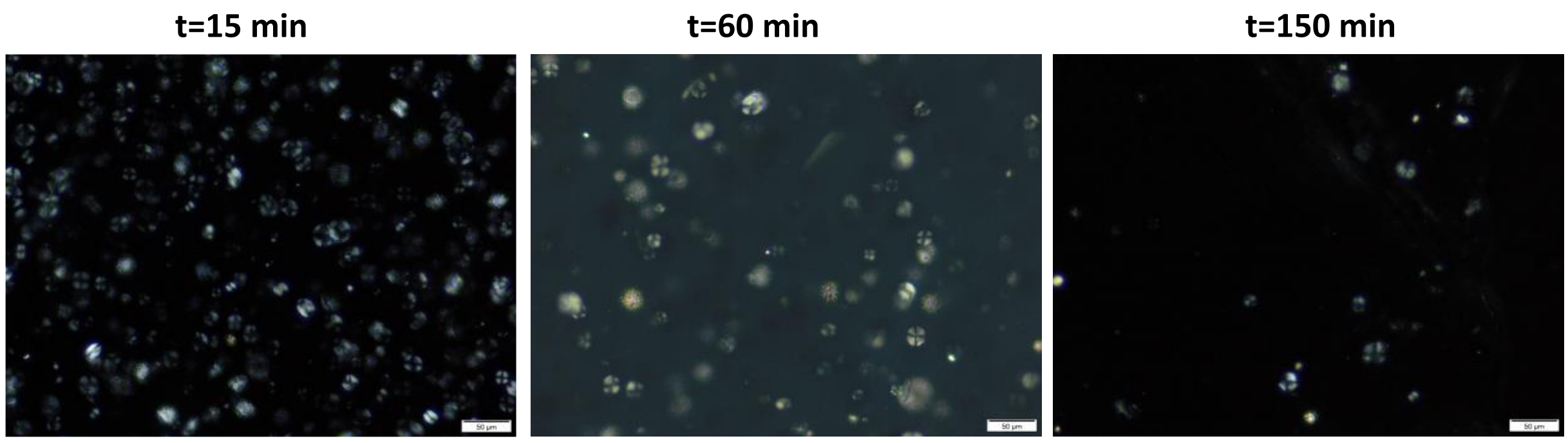

Figure S4. Polarized light optical micrographs of wheat starch granules mixed with St/DES/VL (15/40/45 wt%) after 15-. 60-. and 150-min extrusions at 90 °C.

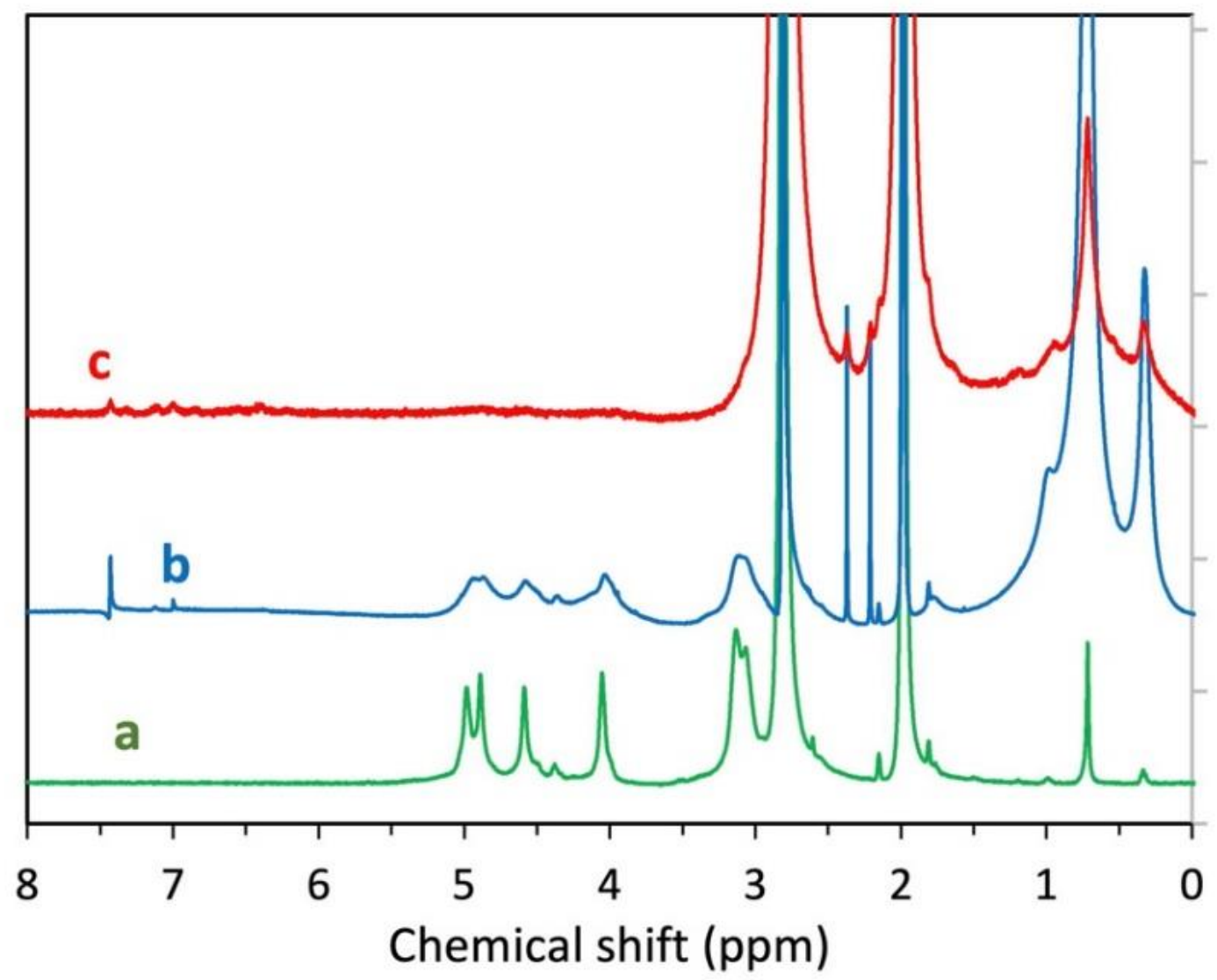

Figure S5. ^{1}H NMR spectra of native starch (a). and VL-esterified starch with VL/GU ratios of 0.7 (b) and 2.0 (c).

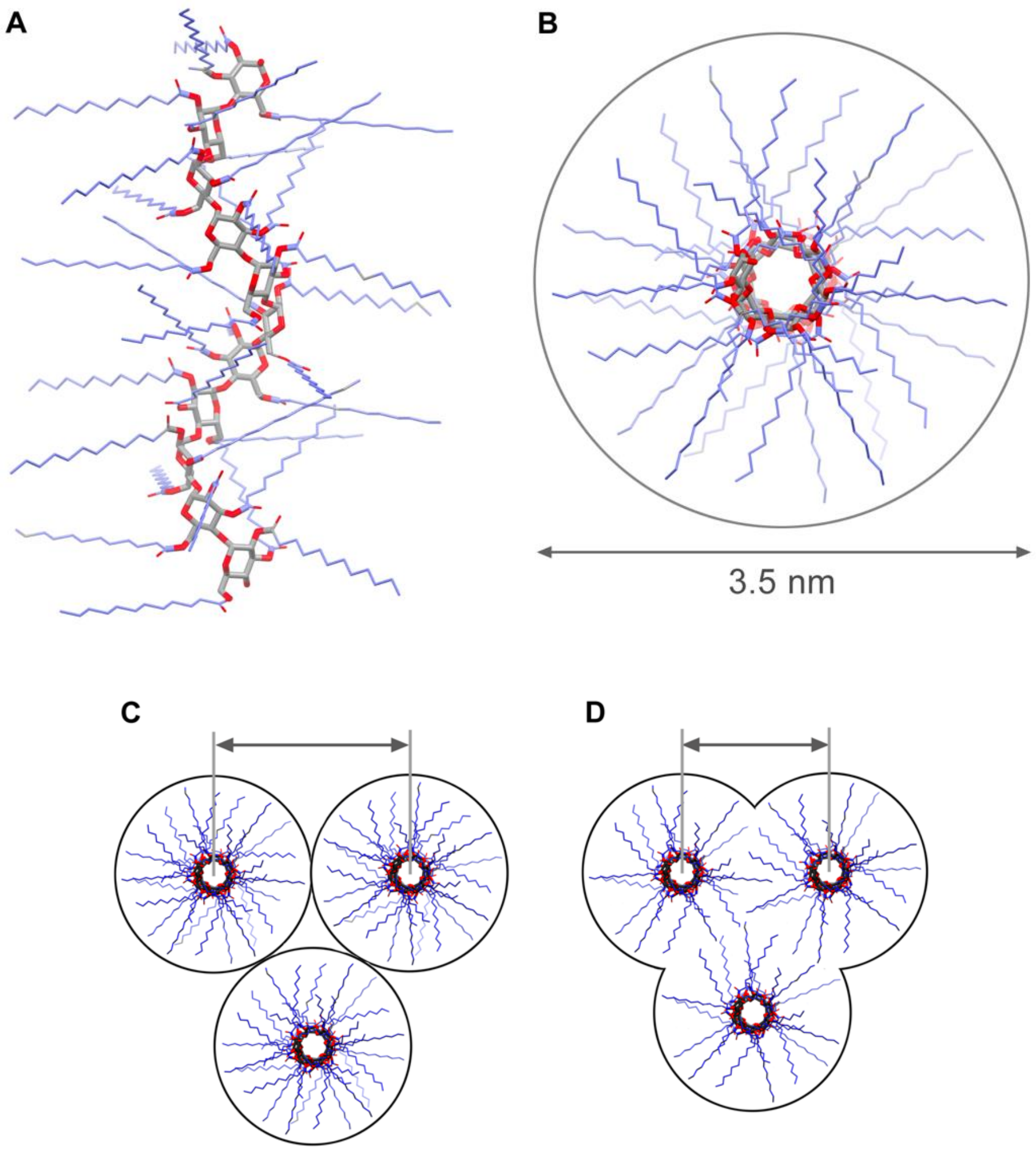

Figure S6. Molecular model of amylose laurate with DS = 3 drawn in longitudinal (A) and axial (B) views with respect to the amylose backbone. The laurate side chains are drawn in blue. Hydrogen atoms were omitted for clarity. Schemes describing the intermolecular distance for DS = 3 (C) and 1< DS < 3 (D). assuming parallel molecules and some degree of interdigitation of the laurate side chains.

The amylose-laurate molecule was constructed using standard bond lengths (CC:1.52 Å. CO: 1.35 Å. CH 1.09 Å. OH: 0.98 Å). bond angles of 109.5°. and standard torsion angles corresponding to -60. 60. and 180° for most torsion angles except for the $H_nC_nOC_L$ and glycosidic angles to avoid steric collision between side chains and high-energy conformation: C3C4OC1: 82°. C4OC1C2: -140°. $C1C2O2C_L$: 130°. C2C3O3C1: -110°. C_L is the first carbon of the lauryl chain at the C=O group.
